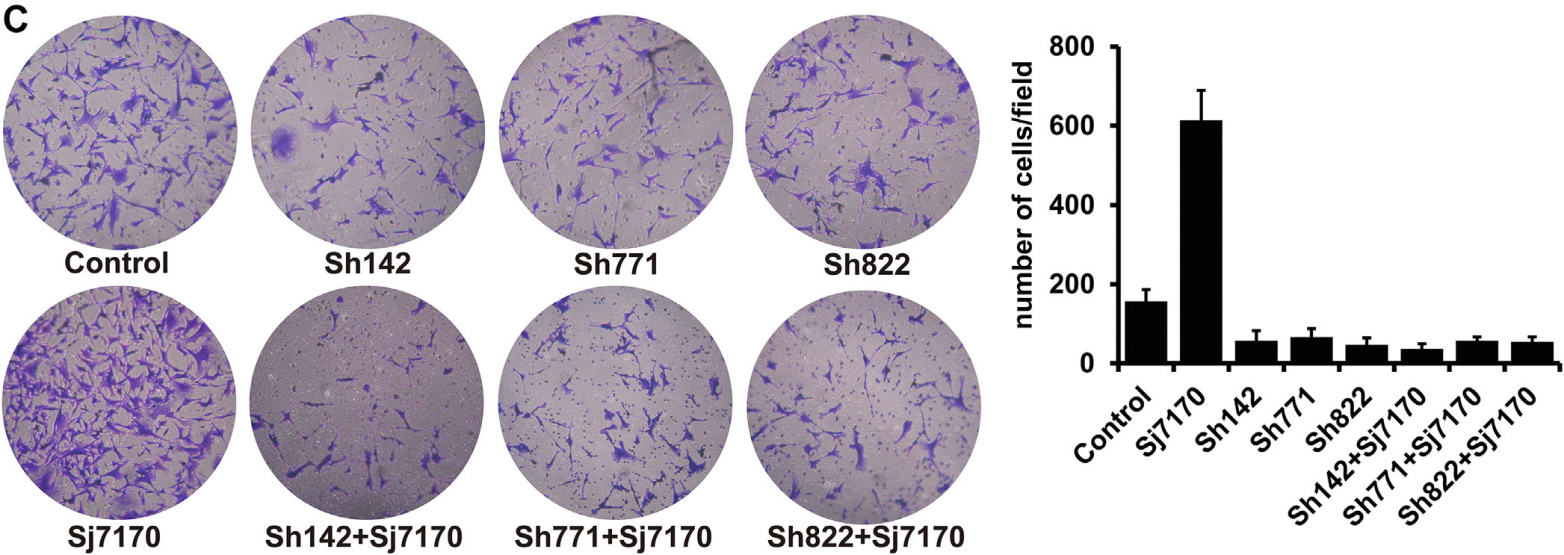# Correction: Sj7170, a unique dual-function peptide with a specific α-chymotrypsin inhibitory activity and a potent tumor-activating effect from scorpion venom

**DOI:** 10.1016/j.jbc.2022.102543

**Published:** 2022-10-21

**Authors:** Yu Song, Ke Gong, Hong Yan, Wei Hong, Le Wang, Yingliang Wu, Wenhua Li, Wenxin Li, Zhijian Cao

Images in Figure 4C and Figure 8C (Sh142+Sj7170 and Sh771+Sj7170) were inadvertently misused. The correct panels of Figure 4C and Figure 8C are shown below. All authors agree that the corrections do not affect the results and conclusions of the article. The authors apologize for those mistakes.

**Figure 4C**

**Figure:**
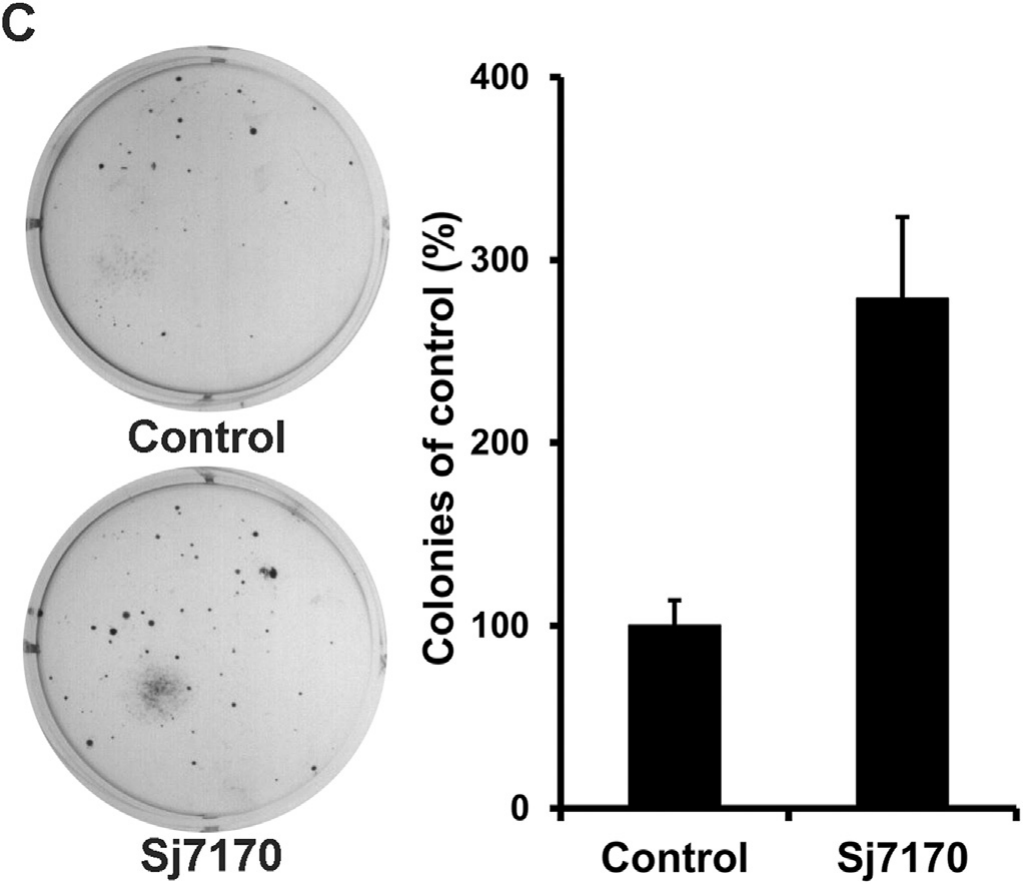

**Figure 8C**

**Figure:**